# Signaling pathway activation drift during aging: Hutchinson-Gilford Progeria Syndrome fibroblasts are comparable to normal middle-age and old-age cells

**DOI:** 10.18632/aging.100717

**Published:** 2015-01-09

**Authors:** Alexander M. Aliper, Antonei Benjamin Csoka, Anton Buzdin, Tomasz Jetka, Sergey Roumiantsev, Alexey Moskalev, Alex Zhavoronkov

**Affiliations:** ^1^ Insilico Medicine, Inc., Johns Hopkins University, ETC, B301, MD 21218, USA; ^2^ Federal Clinical Research Center of Pediatric Hematology, Oncology and Immunology, Moscow, Russia; ^3^ Vision Genomics LLC, Washington, DC 20011, USA; ^4^ Epigenetics Laboratory, Dept. of Anatomy, Howard University, Washington DC 20059, USA; ^5^ Pathway Pharmaceuticals, Limited, 56 Gloucester Rd, Wan Chai, Hong Kong; ^6^ Institute of Fundamental Technological Research, Polish Academy of Sciences, 02-106 Warsaw, Poland; ^7^ Pirogov Russian National Research Medical University, Moscow, 117997, Russia; ^8^ Moscow Institute of Physics and Technology, Dolgoprudny, Moscow Region, 141700, Russia; ^9^ The Biogerontology Research Foundation, BGRF, London W1J 5NE, UK; ^10^ George Mason University, Fairfax, VA 22030, USA

**Keywords:** Progeria, HGPS, Lamin, LMNA, aging, aging pathway, pathway activation, pathway drift, geroscope, geroprotectors, senescence

## Abstract

For the past several decades, research in understanding the molecular basis of human aging has progressed significantly with the analysis of premature aging syndromes. Progerin, an altered form of lamin A, has been identified as the cause of premature aging in Hutchinson-Gilford Progeria Syndrome (HGPS), and may be a contributing causative factor in normal aging. However, the question of whether HGPS actually recapitulates the normal aging process at the cellular and organismal level, or simply mimics the aging phenotype is widely debated. In the present study we analyzed publicly available microarray datasets for fibroblasts undergoing cellular aging in culture, as well as fibroblasts derived from young, middle-age, and old-age individuals, and patients with HGPS. Using GeroScope pathway analysis and drug discovery platform we analyzed the activation states of 65 major cellular signaling pathways. Our analysis reveals that signaling pathway activation states in cells derived from chronologically young patients with HGPS strongly resemble cells taken from normal middle-aged and old individuals. This clearly indicates that HGPS may truly represent accelerated aging, rather than being just a simulacrum. Our data also points to potential pathways that could be targeted to develop drugs and drug combinations for both HGPS and normal aging.

## INTRODUCTION

### Premature aging disorders as models to understand human aging

The complexity of human aging has eluded biologists and physicians for decades, leading to a concerted effort to unravel the physiological, cellular and molecular mechanisms of aging. A potentially successful approach involves the analysis of naturally occurring aging disorders [1,2]. Premature aging is particularly manifested in the rare genetic condition, Hutchinson-Gilford Progeria Syndrome or HGPS [3], which is a disease with major phenotypic features of accelerated cellular, physiological, and anatomical aging of most major tissues and organs [4,5].

The most frequent HGPS mutation is a *de novo* autosomal dominant single base substitution in exon 11 of the *LMNA* gene (C1824T) that activates a cryptic splice site leading to the translation of a truncated lamin-A variant known as Progerin [6]. Progerin remains irreversibly farnesylated and is toxic to cells [7]. Progerin has also been detected in normal individuals throughout their lifespan beginning at 1 month of age [8]; its accumulation leads to DNA damage and is manifested in the molecular response of ATR and ATM activation as well as phosphorylation of Chk1, Chk2 and p53 [9, 10]. Also, fibroblasts from HGPS patients are slower in recruitment of DNA damage response proteins such as p53 binding protein 1(53BP1), thereby indicating defective DNA repair pathways [11].

Lamins A and C, the products of alternate splicing of the *LMNA* gene, are integral components of a dynamic and crucial cellular structure known as the nuclear lamina [12]. They have also been considered as relay platforms for intracellular signaling pathways reaching to the nucleus [13] and interact with chromatin [14,15]. A comparative analysis to map genome-wide interactions of gene promoters with lamin A and Progerin indicates that lamin-A associates with transcription factors, and its variant Progerin induces global changes in chromatin organization by enhancing interactions with a specific subset of genes in addition to defined lamin A associated genes [16].

The biological significance of interactions of lamin A and Progerin-associated genomic regions in terms of aging and disease has remained a major focus of research [17,18]. Genomic instability associated with the defective maturation of prelamin A in HGPS may play a significant role in normal aging and disease [19]. In addition to modulation of DNA repair pathways revealed by studies of HGPS fibroblasts [11], at least a dozen additional pathways have been presumed to induce human aging [20]. These may include alterations in energy metabolism as well as nutrient-sensing pathways [21].

### Does HGPS truly represent accelerated aging, or is it simply a simulacrum?

There has long been a question as to whether HGPS actually recapitulates the normal aging process at the cellular and organismal level, or is simply a simulacrum of aging, that mimics normal aging. However, the pathology of HGPS from the cellular level all the way to the organismal level indicates that almost all of the known characteristics of “normal” aging are recapitulated in HGPS. The major difference may simply be one of cause and effect. What we see as aging, is an “effect” rather than a “cause”; for example shortening of telomeres can be a cause of aging, damage to mitochondria can be a cause of aging, aneuploidy can be a cause of aging, stem cell depletion can be a cause of aging, and so forth [20]. All of these cellular pathologies, and more, can be causative in normal aging, and what we call aging is the *consequence*, or *effect* of these incidents that we define as “cause”. In HGPS, while the primary cause may be overproduction of Progerin, the secondary downstream causes and consequential effects are almost indistinguishable from normal aging, and therefore the condition appears to be very similar or indistinguishable from regular chronological aging.

In a previous comparative microarray analysis we defined a set of 361 genes that showed at least a 2-fold statistically significant alteration in HGPS compared to normal controls; extracellular matrix proteins and transcription factors were the most affected categories, with growth-arrest specific transcription factor MEOX2/GAX being the most significantly affected gene [5]. We have undertaken the current analysis to widen the scope and identify additional pathways commonly affected in aging and premature aging syndromes such as HGPS; in addition, we intend to identify the relevant alterations in these pathways to develop precise therapeutic strategies.

In the present study we have analyzed a wide spectrum of microarray data from replicatively senescent cell lines, cohorts of normally-aging individuals and individuals with HGPS. We have used our newly-developed pathway analysis and drug discovery tool called GeroScope which closely resembles the OncoFinder platform [22,23] to analyze gene expression datasets from multiple platforms with low error rate. This tool has the ability to elucidate and precisely define altered features of intracellular regulation using mathematical computations [24,25]. As an output this algorithm produces Pathway Activation Strength (PAS) values, where positive and negative PAS values indicate pathway up- and down-regulation, respectively. This approach has been previously suggested to investigate the process of aging and rank candidate geroprotectors [26]. On the pathway analysis side the OncoFinder platform was modified to include a collection of signaling pathways implicated in aging and longevity [26].

We divided our analysis into two phases: in phase one we analyzed the pathway changes that occur during increasing cell passage number as part of *in vitro* cellular senescence. Our analysis of replicatively senescing fibroblasts shows that systematic changes in cellular pathway activation states increase as the number of passages increase. We have termed this phenomenon “Pathway Activation Drift”.

In the phase two analysis, we analyzed pathway changes during chronological aging by using data taken from fibroblasts derived from different age groups as well as HGPS patients. Strikingly, this analysis reveals that signaling activation pathways in cells derived from chronologically young patients with HGPS more strongly resemble cells taken from normal middle-aged and old individuals rather than cells taken from young individuals. This strengthens the hypothesis that HGPS truly represents accelerated aging rather than just an aging simulacrum.

Our data also identifies additional affected pathways that show significant therapeutic potential and could be targeted to decelerate the development of normal aging and age-related symptoms using HGPS as a model.

### RESULTS

### Replicative senescence of fibroblasts

In the first phase of our analysis we wished to understand the difference in pathway cloud activation as cells underwent *in vitro* cellular senescence. Therefore we analyzed differences between normal fibroblasts at different *in vitro* cellular passage numbers, and selected the dataset E-MTAB-2086 from the ArrayExpress database (http://www.ebi.ac.uk/arrayexpress/) as source data. Originally derived from a study by Lackner et al. [27], it contains 3 groups of replicatively senescent fibroblasts (cell line IMR90): 50 passages (4 samples), 70 passages (3 samples) and 80 passages (2 samples). Three samples of fibroblasts that underwent only 30 passages were used as reference.

**Figure 1:**
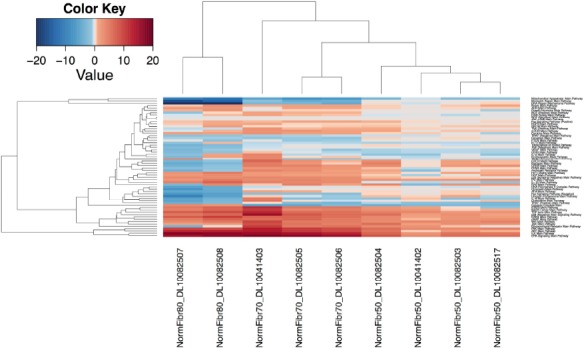
Unsupervised hierarchical clustering heat map of signaling pathways for dataset E-MTAB-2086. Up- and down-regulated pathways are depicted in red and blue color, respectively. The first two clustered samples on the left-hand side of schematic represent the cells with maximum passages of eighty, then follow three samples that underwent 70 passages and four samples grown for 50 passages.

Application of pathway cl oud activation profiles enabled characterization of each group individually at the pathway level (Figure 1) and identification of specific features that distinguished each group based on cellular age. Examples of such pathways include mitochondrial apoptosis, mismatch repair and other DNA-repair mechanisms that are steadily down-regulated with increasing passage number. On the other hand, major pathways such as mTOR, GSK3, TGF-beta, PAK, ILK, GPCR and ERK are steadily up-regulated. Likewise, GPCR, Estrogen and PPAR are up-regulated in groups that underwent 50 and 70 passages, but are then down-regulated after 80 passages. An interesting observation involves an equally mild down-regulation of the FLT3 pathway in all of the samples of two groups of 70 and 50 passages, but variability in the group with 80 passages which exhibits elevated down-regulation in one sample and mild up-regulation in the other. Similarly, SMAD pathway exhibits moderate up-regulation in one 80-passage sample but mild down-regulation in the other. The same pattern is evident in the 70-passage set, where the first sample exhibits moderate up-regulation while the other three exhibit moderate down-regulation. However, mild down-regulation is consistent in all four samples of the 50-passage group. Evidence of variation is fairly consistent in activation and inhibition profiles of cellular-age dependent pathways, and indicates the presence of additional gene and environment-dependent sub-pathways that may differentially cross-react with increasing cellular age.

### Chronological aging of normal and HGPS fibroblasts

Having shown that pathway activation drift occurs during replicative senescence of fibroblasts, we decided to compare signaling pathway activation of progeric HGPS fibroblasts and fibroblasts taken from healthy individuals of different ages. Taken from 7 different publicly available datasets, based on the donor's age, all of the samples were divided into four groups: “Young”, “Middle”, “Old” and “HGPS” fibroblasts (Table 1). PAS values were obtained for each individual sample of each group.

**Table 1:** Numbers of analyzed samples divided into four investigated groups (“Young”, “Middle”, “Old” and “HGPS”)

Dataset identifier	Young	Middle	Old	HGPS	Reference
GSE3860	-	-	-	18	[50]
GSE15829	-	1	4	-	[51]
GSE17032	4	19	1	-	[52]
GSE28300	6	-	6	-	[53]
GSE55118	5	5	5	-	[54]
E-MEXP-2597	-	-	-	5	[55]
E-MEXP-3097	-	-	-	3	[56]
Total	15	25	16	26	

**Figure 2:**
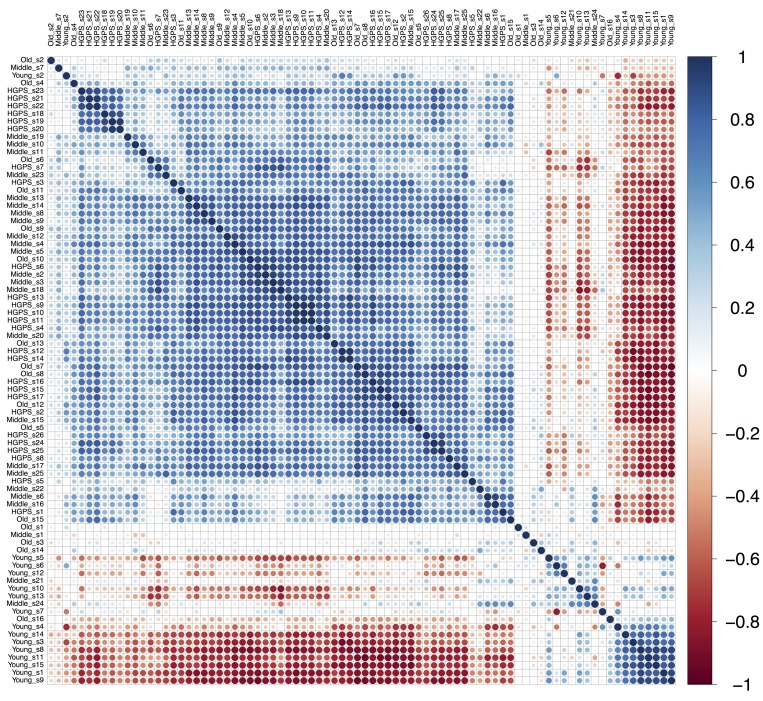
Pearson's correlation plot build for “Young”, “Middle”, “Old” and “HGPS” groups of fibroblasts. Samples from all datasets are combined and named according to the group they belong to. Scale bar colors indicate the sign and magnitude of Pearson's correlation coefficient between samples.

**Figure 3:**
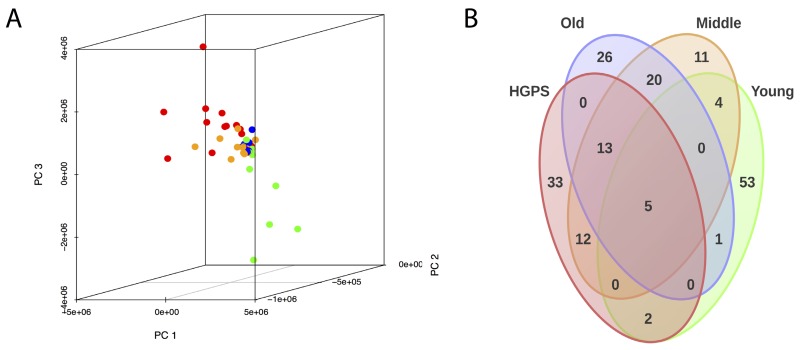
(**A**) PAS values of all samples were transformed into the first three principal components using principal component analysis (PCA). “Young”, “Middle”, “Old” and “HGPS” are depicted in green, yellow, blue and red color respectively. (**B)** Venn diagram representing the number of similarly up-/down-regulated pathways between “Young”, “Middle”, “Old” and “HGPS” groups. Similarity of PAS values distributions of different pathways for given groups were computed using equivalence T-test (pairwise comparison) and equivalence F-test (comparison of three and four groups).

Comparative Pearson's correlation shows a clear similarity between “HGPS”, “Middle” and “Old” groups (Figure 2). Apart for several outliers, the samples in these three groups are tightly clustered and positively correlated with each other. On the other hand, the samples from the “Young” group show negative correlation with the three other groups. Principal component analysis also shows that “HGPS”, “Middle” and “Old” groups are closely allocated, even though “HGPS” samples have a higher variability and in some respect they are closer to the “Middle” group (Figure 3, A). Surprisingly, derived from different datasets samples from the “Old” group form a very compact cluster. In contrast, one third of the “Young” group samples have high pathway activation variability and they cluster apart from the other groups. We also produced a Venn diagram representing the number of similarly up-/down-regulated pathways between “Young”, “Middle”, “Old” and “HGPS” groups (Figure 3, B). Similarity of PAS values distributions of different pathways for given groups were computed using equivalence T-test for pairwise comparison and equivalence F-test for comparison of three and four groups. In accordance with previous findings “HGPS”, “Middle” and “Old” groups have 13 similarly activated signaling pathways. Among these AR, IGF1R, HGF, HIF1A, IP3, PAK, SMAD, TNF and TGF-beta main pathways are up-regulated, whereas Mitochondrial apoptosis main pathway is down-regulated. Interestingly, pairwise comparison between “HGPS” and “Middle” groups showed 12 additional similarly activated pathways, once again implying their closeness on the signalome level. On the other hand, most of the signaling pathways for the “Young” group are not similarly activated compared with the rest of investigated groups. Of note, there're 5 signaling pathways mostly associated with DNA repair that are similarly deviating around zero in all four investigated groups.

## DISCUSSION

Our new data analysis clearly demonstrates that the process of aging and the pathophysiology of the premature aging syndrome HGPS are biological processes regulated through similar pathways; each phenomenon has intricate control mechanisms that are distinct yet interrelated. There is clear indication of signaling disturbance evidenced by the altered expression of specific pathways; the major impact is manifested in pathways that affect DNA repair and chromatin organization in particular.

Quantitatively diverse gene expression patterns are suggestive of distinct and strict patterns of regulatory and structural proteins that participate in the development of aging symptoms at the cellular level, as well as at the level of the whole organism. Such qualitative variability in gene expression is most likely based on genetic background, whereas the resulting pathology derives from the interaction between the genome and epigenome with environmental factors and physiological stress.

### Summary of significantly altered pathways

As organismal aging affects the entire body, it is likely to influence a number of pathways simultaneously. How-ever, the degree of impact has to be variable depending on the tissue in question, as well as living conditions of the organism and unexpected environmental stress. Below we present examples of significantly altered path-ways that are clearly evident in our data analysis, which substantiates the previously characterized findings and reveals the correlation of cellular and organismal aging.

### Caspase cascade pathway

**Figure 4:**
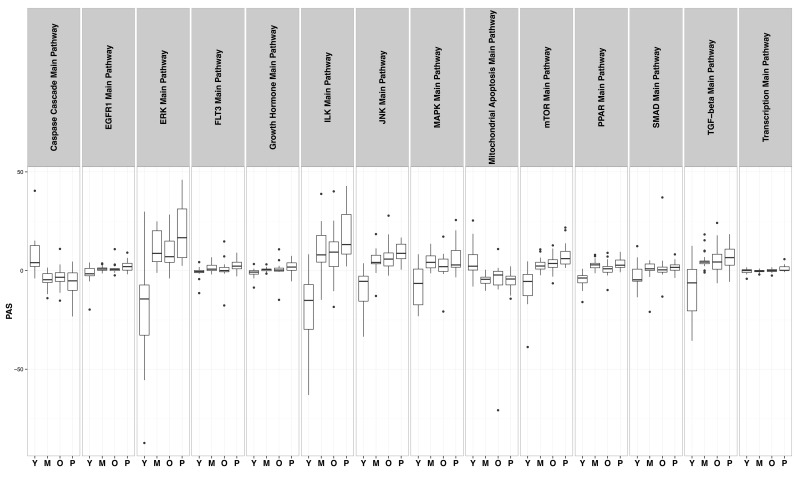
Distribution of PAS values in “Young” (Y), “Middle” (M), “Old” (O) and “HGPS” (P) groups in 14 different signaling pathways.

The first pathway that shows clear alteration in cells with increasing number of passages is the caspase cascade pathway; indeed cells with additional passages show a caspase expression profile akin to Progeric cells (Figure 4, panel 1). Furthermore, experimental evidence in mice indicates that caspase-2 deficiency enhances aging traits [28]; our data indicate down regulation of caspase pathway in aging fibroblasts.

### EGFR pathway

In the course of normal wound healing, fibroblasts from surrounding intact tissue are recruited to the site of injury/wound where they proliferate and regenerate to participate in tissue repair and wound healing; however, their proliferative and migratory abilities are age-dependent showing a significant reduction as a function of age [29]. Further analysis to identify the underlying molecular mechanism of the phenomenon detected reduced responsiveness to epidermal growth factor (EGF) due to preferential loss of EGF receptors in these cells [30]. Panel 2 in Figure 4 shows the aging effect through cell-passages on EGFR1 mediated pathways where cells representing older age show a profile closely related to HGPS cells and the same trend is evident in cells representing middle age. In contrast, youthful cells show a completely opposite activation profile.

### ERK pathway

Dietary restriction through alternate day fasting has been shown to reverse the age-associated cardiac hypertrophy phenotype in rats through alteration of ERK and P13K signaling pathways [31]. In our pathway analysis, cells representing middle and old age individuals show a tendency of activation of ERK pathway in the same manner as has been observed in cells from HGPS patients where cardiac phenotype is the major cause of premature death. The activation profile is completely opposite to the one observed in young cells (Figure 4, panel 3).

### Growth hormones and mediator associated pathways

Mammalian growth hormone is produced in the anterior pituitary, but its secondary mediator insulin-like growth factor (IGF-1) is produced in diverse cell types and has the same signaling pathway as insulin [32] thereby participating in nutrient sensing or more specifically in detecting the presence of glucose. This pathway has been shown to be activated in HGPS fibroblasts, and our aging cells of both middle and older age groups show a similar but slightly weaker trend of activation as seen in panel 5 of Figure 3; the same is true for the FLT3 pathway as shown in panel 4. For this particular pathway, both the middle and older aged group of cells show very similar profiles of activation. In vivo administration of hematopoietic growth factor FLT3 ligand (FLT3L) has been shown to reverse age-associated defects in the thymus of nude mice [33] and therefore, it is reasonable to assume that a similar mechanism is at work in cultured mammalian cells representing different stag es of mammalian aging.

### MAPK pathway

MAPK pathway has been found to increase p38 phosphorylation in aging mice. It is the key regulator of proinflammatory response in aging cells [34]. In our pathway analysis, alteration in MAPK pathways in middle-age and old samples resembles the profile evident in HGPS cells; the profile in young cells is distinct from these three cell types.

### Mitochondrial dysfunction and associated pathways

Mitochondrial dysfunction has been associated with sarcopenia, an age-related decline of skeletal muscle mass and function [35]. In addition to their participation in the life and death of the cell through ATP production, mitochondria regulate cellular apoptosis through both extrinsic and intrinsic pathways [36]. In our analysis, both the older and middle-aged groups of cells show activation of mitochondria mediated pathways with the same trend as progeric cells, despite a slightly milder overall impact.

### mTOR pathway

The mTOR pathway is vital for multiple cellular activities [37] and is involved in increasing longevity; its inhibition resembles dietary restriction [38]. Similar to Progeric cells, cells equivalent to both middle and old age groups show activation of the mTOR pathway; activation is greater in older cells than in middle age cells. However, the activation profile is still slightly lower than HGPS cells, which represent not only accelerated aging, but a highly reduced longevity.

### Nuclear receptor mediated PPAR pathway

Nuclear receptors that belong to the group of peroxisome-proliferator activated receptors (PPAR) participate in key cellular processes of differentiation, development, metabolism and malignancy through regulation of gene transcription [39]. HGPS cells clearly show upregulation of the PPAR pathway, and our cell groups of increased passages representing middle and old age respectively also show PPAR pathway activation above the threshold level observed in young cells. However, in this particular case the group representing middle age also shows significant impact.

### SMAD-associated pathways

Paracrine mechanisms involving TGF-β and its receptors as well as other cytokines are involved in skin aging as well as treatment of UV induced damage [40]. Initiation of TGF-β signaling occurs with the binding of a growth factor molecule with its receptor, either TGF-β receptor type II, a serine-threonine kinase molecule. This ligand bound complex is involved in activation of TGF-β receptor type I through its phosphorylation; the activated molecule propagates the signal through phosphorylation of intracellular proteins known as Smad proteins that translocate the activated complex to the nucleus to participate in transcriptional regulation of target genes [41,42]. The twelfth panel of Figure 4 shows the activation profile of the Smad group of proteins in the pathway with a striking similarity shown between older cells and HGPS cells; in cells representing middle age, activation follows the same trend but is slightly weaker despite being distinctly different from the profile evident in younger cells.

### TGF-β-associated pathway

Differential TGF-β signaling has been found to be associated with skin conditions of aged ethnic groups, leading to specific aging skin features [42]. Our comparative pathway analysis reveals that alteration in TGF-β signaling pathway in older cells closely resembles that in progeric cells that are severely affected in patients with HGPS.

### Transcription and associated pathways

Translational modulation is another pathway likely affected in an age-dependent manner, leading to altered protein expression. Global association of lamin A with transcriptional factors and participation of progerin in alteration of chromatin organization are strong indicators of putative alteration in transcription and associated pathways in aging. This indeed is evident in the last panel of Figure 4 for cells representing middle and old age; the differential alteration in pHGPS cells is clearly evident as well. Therefore, the increasing number of cellular passage appears to recapitulate organismal aging at the cellular level.

### Conclusions

Key findings of various altered pathways outlined above are consistent with previously characterized gene expression alterations in various cell lines, primary cultures of HGPS patients as well as those from normally aging control individuals. The data presented here provide evidence that cellular aging is the pre-condition for anatomical aging, and the overall aging phenotype of the organism is the final outcome of cellular aging mediated through the aging features in specific tissues. Similarly, the comparative analysis of altered pathways in the cells derived from HGPS patients with normal fibroblasts representing two different age groups indicates a higher order of magnitude for the every altered pathway in HGPS cells. Based on these observations, we can safely conclude that HGPS indeed represents accelerated aging and is not simply a “simulacrum” for aging.

Since all of these disorders are monogenic, it may be possible to find defensive or common pathways that cause universal aging phenotypes resulting from gradual misregulation as a function of age along with environmental factors. Several pharmaceutical drugs are likely to affect age-associated disorders [43,44] as well as aging and progeria [45]. Many drugs have known effects on gene expression in the various cells and tissues [46], and this information could be utilized for in silico screening of possible geroprotectors and their combinations. This study also strengthens the hypothesis that rapamycin, everolimus and analogues acting on the mTOR pathway may act as possible geroprotectors effective in HGPS and normal aging [45,46].

In order to define the specific steps of molecular modulation of the pathways that lead to HGPS, in future work we intend to extend our comparative analysis to aging, HGPS and Werner's Syndrome (WS), a condition where accelerated aging begins near puberty. In addition, we shall evaluate other maladies including Bloom syndrome (BS), Rothmund–Thomson syndrome (RTS), Cockayne syndrome (CS), xeroderma pigmentosum (XP), trichothiodystrophy (TTD), combined xeroderma pigmentosum-Cockayne syndrome (XP-CS) and restrictive dermopathy (RD) in order to identify common PAS patterns.

## METHODS

In this study we analysed the samples gathered from different datasets found in Gene Expression Omnibus (GEO) and ArrayExpress database (http://www.ebi.ac.uk/arrayexpress/). To investigate the replicative senescence of fibroblasts we used the dataset E-MTAB-2086 by Lackner et al. [27]. For the comparison of young, middle age, old and HGPS fibroblasts we utilized 7 different datasets (summariz-ed in Table 1).

All preprocessing procedures were performed using R (http://www.R-project.org/) and Bioconductor Project [47,48]. Raw microarray data from dataset E-MTAB-2086 was preprocessed and quantile normalized using “oligo” package [49]. Raw microarray data from datasets GSE3860 [50], GSE15829 [51], GSE17032 [52], E-MEXP-2597 [55] and E-MEXP-3097 [56] was normalized using a cytosine guanine robust multi-array analysis (GCRMA) algorithm and summarized using updated chip definition files from Brainarray repository (Version 18) [57]. Datasets GSE28300 [53] and GSE55118 [54] were preprocessed using “limma” package from Bioconductor, implementing ‘normexp’ background correction and quantile normalization [58]. Due to the absence of the raw data, preprocessed gene expression values for dataset GSE15829 [51] were taken as provided by authors in the original GEO record.

All of the samples gathered from these datasets were divided into four groups: young, middle, old and HGPS fibroblasts. The “Young”, “Middle”, “Old” groups were formed according to the donor's age. “Young” group consisted of skin fibroblasts collected from donors of 15-30 years old, “Middle” - 40-55 y.o., “Old” - 60+ y.o.

Each preprocessed gene expression dataset was independently analyzed using an algorithm called OncoFinder [22] implemented in a new platform for analyzing signaling pathways in aging called GeroScope. Taking the preprocessed gene expression data as an input it allows for cross-platform dataset comparison with low error rate and has the ability to obtain functional features of intracellular regulation using mathematical estimations. For each investigated sample it performs a case-control pairwise comparison using Student's t-test, generates the list of significantly differentially expressed genes and calculates the Pathway Activation Strength (PAS), a value which serves as a qualitative measure of pathway activation. Positive and negative PAS values indicate pathway up- and down-regulation, respectively. In this study the genes with ∣fold-change∣≥1.5 and FDR-adjusted p-value<0.05 were considered significantly differentially expressed.

Each dataset was processed separately and PAS values for 65 main signaling pathways were calculated for every investigated sample in the dataset (Supplementary table 1). The PAS values for “HGPS” group from datasets GSE3860 [50], E-MEXP-2597 [55] and E-MEXP-3097 [56] were acquired using healthy human fibroblasts taken from these datasets as a reference. In turn, the PAS values for “Young”, “Middle” and “Old” groups from datasets were obtained from datasets GSE15829 [51], GSE17032 [52], GSE28300 [53] and GSE55118 [54], using the samples from “Young” group as a reference. Since the “Young” group samples was normalized on itself, all PAS values of all individual samples were zero when standard filtering thresholds were applied. However, to account for any pathway activation variation and gather more information, we decided to process the “Young” group without filtering and account the expression level of all genes obtaining non-zero PAS values for individual samples. After PAS values for all datasets had been calculated all samples were combined into the group they belong to, resulting in 15, 25, 16 and 26 samples in “Young”, “Middle”, “Old” and “HGPS” groups respectively (Table 1).

Hierarchical clustering heatmaps were generated using ‘heatmap.2‘ function from ‘gplots’ package [59]. Pearson's correlation plot with hierarchical clustering of samples was built using ‘corrplot’ package [60]. Principal component analysis was done using the ‘prcomp’ function in the ‘stats’ package. For determining similarities of pathways between investigated groups statistical equivalence tests were conducted. They test the null hypothesis of difference in distribution of PAS value between conditions against the alternative hypothesis that these distributions are similar. For pairwise comparison of two conditions, an equivalence t-test was used. For comparison of three and four conditions we performed an equivalence F-test (equivalence one-way ANOVA test). Equivalence intervals were chosen according to referenced values from [61], so that all tests are comparable (0.74 for t-test and 0.74/sqrt(2) for F-test). Significance was established using significance level 0.05.

## SUPPLEMENTARY TABLE



## References

[1] Misteli T (2011). HGPS-Derived iPSCs For The Ages. Cell Stem Cell.

[2] Cadiñanos J, Ignacio V, López-Otín C (2005). Perspective From Immature Lamin to Premature Aging. Cell Cycle.

[3] Dreesen O, Stewart CL (2011). Accelerated aging syndromes, are they relevant to normal human aging?. Aging (Albany NY).

[4] DeBusk FL (1972). The Hutchinson-Gilford progeria syndrome. J. Pediatr. CRC Press.

[5] Csoka AB, English SB, Simkevich CP, Ginzinger DG, Butte AJ, Schatten GP, Rothman FG, Sedivy JM (2004). Genome-scale expression profiling of Hutchinson-Gilford progeria syndrome reveals widespread transcriptional misregulation leading to mesodermal/mesenchymal defects and accelerated atherosclerosis. Aging Cell.

[6] Eriksson M, Brown WT, Gordon LB, Glynn MW, Singer J, Scott L, Erdos MR, Robbins CM, Moses TY, Berglund P, Dutra A, Pak E, Durkin S (2003). Recurrent de novo point mutations in lamin A cause Hutchinson-Gilford progeria syndrome. Nature.

[7] De Sandre-Giovannoli A, Bernard R, Cau P, Navarro C, Amiel J, Boccaccio I, Lyonnet S, Stewart CL, Munnich A, Le Merrer M, Levy N (2003). Lamin A truncation in Hutchinson-Gilford progeria. Science.

[8] Olive M, Harten I, Mitchell R, Beers JK, Djabali K, Cao K, Erdos MR, Blair C, Funke B, Smoot L, Gerhard-Herman M, Machan JT, Kutys R (2010). Cardiovascular pathology in Hutchinson-Gilford progeria: correlation with the vascular pathology of aging. Arterioscler Thromb Vasc Biol.

[9] Liu Y, Rusinol A, Sinensky M, Wang Y, Zou Y (2006). DNA damage responses in progeroid syndromes arise from defective maturation of prelamin A. J Cell Sci.

[10] Varela I, Cadiñanos J, Pendás AM, Gutiérrez-Fernández A, Folgueras AR, Sánchez LM, Zhou Z, Rodríguez FJ, Stewart CL, Vega JA, Tryggvason K, Freije JM, López-Otín C (2005). Accelerated ageing in mice deficientin Zmpste24 protease is linked to p53 signalling activation. Nature.

[11] Liu B, Wang J, Chan KM, Tjia WM, Deng W, Guan X, Huang JD, Li KM, Chau PY, Chen DJ, Pei D, Pendas AM, Cadiñanos J (2005). Genomic instability in laminopathy-based premature aging. Nat Med.

[12] Kolb T, Maass K, Hergt M, Aebi U, Herrman H (2011). Lamin, A, and lamin C form homodimers and coexist in higher complex forms both in the nucleoplasmic fraction and in the lamina of cultured human cells. Nucleus.

[13] Dauer WT, Worman HJ (2009). The nuclear envelope as a signaling node in development and disease. Dev Cell.

[14] Dechat T, Adam SA, Goldman RD (2009). Nuclear lamins and chromatin: when structure meets function. Adv Enzyme Regul.

[15] Kind J, van Steensel B (2010). Genome-nuclear lamina interactions and gene regulation. Curr Opin Cell Biol.

[16] Kubben N, Adriaens M, Meuleman W, Voncken JW, van Steensel B, Misteli T (2012). Mapping of lamin A- and progerin-interacting genome regions. Chromosoma.

[17] Collas P, Lund EG, Oldenburg AR (2014). Closing the (nuclear) envelope on the genome: how nuclear lamins interact with promoters and modulate gene expression. Bioessays.

[18] Pegoraro G, Misteli T (2009). The central role of chromatin maintenance in aging. Aging (Albany NY).

[19] Musich PR, Yue Z (2009). Genomic instability and DNA damage res ponses in progeria arising from defective maturation of prelamin A. Aging (Albany NY).

[20] Fontana L, Kennedy BK, Longo VD (2014). Prepare for human testing. Nature.

[21] Fontana L, Partridge L, Longo VD (2010). Extending healthy life span — from yeast to humans. Science.

[22] Buzdin AA, Zhavoronkov AA, Korzinkin MB, Venkova LS, Zenin AA, Smirnov PY, Borisov NM (2014). Oncofinder, a new method for the analysis of intracellular signaling pathway activation using transcriptomic data. Front Genet.

[23] Buzdin AA, Zhavoronkov AA, Korzinkin MB, Roumiantsev SA, Aliper AM, Venkova LS, Smirnov PY, Borisov NM (2014). The OncoFinder algorithm for minimizing the errors introduced by the high-throughput methods of transcriptome analysis. Front. Mol. Biosci.

[24] Borisov NM, Terekhanova NV, Aliper AM, Venkova LS, Smirnov PY, Roumiantsev SA, Korzinkin MB, Zhavoronkov AA, Buzdin AA (2014). Signaling pathways activation profiles make better markers of cancer than expression of individual genes. Oncotarget.

[25] Spirin PV, Lebedev TD, Orlova NN, Gornostaeva AS, Prokofjeva MM, Nikitenko NA, Dmitriev SE, Buzdin AA, Borisov NM, Aliper AM, Garazha AV, Rubtsov PM, Stocking C, Prassolov VS (2014). Silencing AML1-ETO gene expression leads to simultaneous activation of both pro-apoptotic and proliferation signaling. Leukemia.

[26] Zhavoronkov AA, Buzdin AA, Garazha AV, Borisov NM, Moskalev AA (2014). Signaling pathway cloud regulation for in silico screening and ranking of the potential geroprotective drugs. Front Genet.

[27] Lackner DH, Hayashi MT, Cesare AJ, Karlseder J (2014). A genomics approach identifies senescence-specific gene expression regulation. Aging Cell.

[28] Zhang Y, Padalecki SS, Chaudhuri AR, De Waal E, Goins BA, Grubbs B, Ikeno Y, Richardson A, Mundy GR, Herman B (2007). Caspase-2 deficiency enhances aging-related traits in mice. Mechanisms of Ageing and Development.

[29] Ashcroft GS, Horan MA, Ferguson MW (1995). The effects of aging on cutaneous wound-healing in mammals. J Anat.

[30] Shiraha H, Gupta K, Drabik K, Wells A (2000). Aging fibroblasts present reduced epidermal growth factor (EGF) responsiveness due to preferential loss of EGF receptors. J Biol Chem.

[31] Castello L, Maina M, Testa G, Cavallini G, Biasi F, Donati A, Leonarduzzi G, Bergamini E, Poli G, Chiarpotto E (2011). Alternate-day fasting reverses the age-associated hypertrophy phenotype in rat heart by influencing the ERK and PI3K signaling pathways. Mechanisms of Ageing and Development.

[32] López-Otín C, Blasco MA, Partridge L, Serrano M, Kroemer G (2013). The hallmarks of aging. Cell.

[33] Shurin GV, Chatta GS, Tourkova IL, Zorina TD, Esche C, Shurin MR (2004). Regulation of dendritic cell expansion in aged athymic nude mice by FLT3 ligand. Experimental Gerontology.

[34] Li Z, Li J, Bu X, Liu X, Tankersley CG, Wang C, Huang K (2011). Age-induced augmentation of p38 MAPK phosphorylation in mouse lung. Experimental Gerontology.

[35] Marzetti E, Calvani R, Cesari M, Buford TW, Lorenzi M, Behnke BJ, Leeuwenburgh C (2013). Mitochondrial dysfunction and sarcopenia of aging: From signaling pathways to clinical trials. The International Journal of Biochemistry & Cell Biology.

[36] Mammucari C, Rizzuto R (2010). Signaling pathways in mitochondrial dysfunction and aging. Mechanisms of Ageing and Development.

[37] Houédé N, Pourquier P (2014). Targeting the genetic alterations of the PI3K-AKT-mTOR pathway: Its potential use in the treatment of bladder cancers. Pharmacology & Therapeutics.

[38] Johnson TE (2013). Rapid Aging Rescue?. Science.

[39] Feige JN, Gelman L, Michalik L, Desvergne B, Wahli W (2006). From molecular action to physiological outputs: Peroxisome proliferator-activated receptors are nuclear receptors at the crossroads of key cellular functions. Prog. Lipid Res.

[40] Rittie L, Fisher GJ (2002). UV-light-induced signal cascades and skin aging. Ageing Res. Rev.

[41] Massague J (1998). TGF-b signal transduction. Annu Rev Biochem.

[42] Han KH, Choi HR, Won CH, Chung JH, Cho KH, Eun HC, Kim KH (2005). Alteration of the TGF-B/SMAD pathway in intrinsically and UV-induced skin aging. Mechanisms of Ageing and Development.

[43] Berstein LM (2012). Metformin in obesity, cancer and aging: addressing controversies. Aging (Albany NY).

[44] Halicka HD, Hong Z, Jiangwei L, Yong-Syu L, Tze-Chen H, Wu JM, Zbigniew D (2012). Potential anti-aging agents suppress the level of constitutive mTOR-and DNA damage-signaling. Aging (Albany NY).

[45] Blagosklonny MV (2011). Progeria, rapamycin and normal aging: recent breakthrough. Aging (Albany NY).

[46] Shor B, Gibbons JJ, Abraham RT, Yu K (2009). Targeting mTOR globally in cancer: thinking beyond rapamycin. Cell Cycle.

[47] Gentleman RC, Carey VJ, Bates DM, Bolstad B, Dettling M, Dudoit S, Ellis B, Gautier L, Ge Y, Gentry J, Hornik K, Hothorn T (2004). Bioconductor: open software development for computational biology and bioinformatics. Genome Biol.

[48] Carey VJ, Gentry J, Whalen E, Gentleman R (2005). Network structures and algorithms in Bioconductor. Bioinformatics.

[49] Carvalho BS, Irizarry RA (2010). A framework for oligonucleotide microarray preprocessing. Bioinformatics.

[50] Csoka AB, English SB, Simkevich CP, Ginzinger DG, Butte AJ, Schatten GP, Rothman FG, Sedivy JM (2004). Genome-scale expression profiling of Hutchinson-Gilford progeria syndrome reveals widespread transcriptional misregulation leading to mesodermal/mesenchymal defects and accelerated athero-sclerosis. Aging Cell.

[51] Kriete A, Mayo KL, Yalamanchili N, Beggs W, Bender P, Kari C, Rodeck U (2008). Cell autonomous expression of inflammatory genes in biologically aged fibroblasts associated with elevated NF-kappaB activity. Immun Ageing.

[52] Wadlow RC, Wittner BS, Finley SA, Bergquist H, Upadhyay R, Finn S, Loda M, Mahmood U, Ramaswamy S (2009). Systems-level modeling of cancer-fibroblast interaction. PLoS One.

[53] Dekker P, Gunn D, McBryan T, Dirks RW, van Heemst D, Lim FL, Jochemsen AG, Verlaan-de Vries M, Nagel J, Adams PD, Tanke HJ, Westendorp RG, Maier AB (2012). Microarray-based identification of age-dependent differences in gene expression of human dermal fibroblasts. Mech Ageing Dev.

[54] Kalfalah F, Sobek S, Bornholz B, Götz-Rösch C, Tigges J, Fritsche E, Krutmann J, Köhrer K, Deenen R, Ohse S, Boerries M, Busch H, Boege F (2014). Inadequate mito-biogenesis in primary dermal fibroblasts from old humans is associated with impairment of PGC1A-independent stimulation. Exp Gerontol.

[55] Marji J, O'Donoghue SI, McClintock D, Satagopam VP, Schneider R, Ratner D, Worman HJ, Gordon LB, Djabali K (2010). Defective lamin A-Rb signaling in Hutchinson-Gilford Progeria Syndrome and reversal by farnesyltransferase inhibition. PLoS One.

[56] Plasilova M, Chattopadhyay C, Ghosh A, Wenzel F, Demougin P, Noppen C, Schaub N, Szinnai G, Terracciano L, Heinimann K (2011). Discordant gene expression signatures and related phenotypic differences in lamin A- and A/C-related Hutchinson-Gilford progeria syndrome (HGPS). PLoS One.

[57] Dai C, Liu J (2005). Inducing Pairwise Gene Interactions from Time-Series Data by EDA Based Bayesian Network. Conf Proc. Annu Int Conf IEEE Eng Med Biol Soc.

[58] Smyth GK, Gentleman R, Carey V, Dudoit S, Irizarry R, Huber W (2005). Limma: linear models for microarray data. ‘Bioinformatics and Computational Biology Solutions using R and Bioconductor’.

[59] Warnes GR, Bolker B, Bonebakker L, Gentleman R, Lumley WT, Maechler M, Magnusson A, Moeller S, Schwartz M, Venables B (2014). gplots: Various R programming tools for plotting data.

[60] Wei T (2013). corrplot: Visualization of a correlation matrix. http://CRAN.R-project.org/package=corrplot.

[61] Wellek S (2010). Testing statistical hypotheses of equivalence and noninferiority.

